# Biosecurity and Management Strategies for Economically Important Exotic Tephritid Fruit Fly Species in Australia

**DOI:** 10.3390/insects14100801

**Published:** 2023-10-04

**Authors:** Jessica L. Hoskins, Polychronis Rempoulakis, Mark M. Stevens, Bernard C. Dominiak

**Affiliations:** 1 Yanco Agricultural Institute, New South Wales Department of Primary Industries, Private Mail Bag, Yanco, NSW 2703, Australia; mark.stevens@dpi.nsw.gov.au; 2 Central Coast Primary Industries Centre, New South Wales Department of Primary Industries, Locked Bag 26, Gosford, NSW 2250, Australia; polychronis.rempoulakis@dpi.nsw.gov.au; 3 The Ian Armstrong Building, New South Wales Department of Primary Industries, 105 Prince Street, Orange, NSW 2280, Australia; bernie.dominiak@dpi.nsw.gov.au

**Keywords:** incursion, management, emergency response, *Bactrocera dorsalis*

## Abstract

**Simple Summary:**

Australian horticulture is at risk from incursions by exotic tephritid fruit fly species, particularly oriental fruit fly. An incursion by exotic fruit flies is likely to result in global trade restrictions and will cause significant economic losses. In this study, we investigated global management strategies for exotic fruit fly species and compared them with available local strategies in Australia to identify areas where Australian management practices could be improved. We identified that although Australia has a good understanding of the main exotic fruit fly threats, there remains no species-specific emergency response plan available to implement in the case of an incursion. Australia has effective tools for exotic fruit fly detection, an early warning surveillance network is in place across Australia and control measures used successfully for eradication elsewhere are available in Australia. However, the speed at which Australia could respond to an incursion is currently limited by the lack of a response plan, and this could have major implications for the effectiveness of management strategies for containment and eradication, likely leading to a more severe and costly incursion outcome.

**Abstract:**

Exotic tephritid incursions are of high concern to Australia’s biosecurity and its horticultural industries. It is vital that Australia remains ready to respond to incursions as they arise, as an incursion of tephritid fruit fly species will result in significant economic losses. In this review, we compared Australian incursion management strategies for fruit flies with global management strategies and identified possible areas where improvements could be made in an Australian context. Overall, Australia has a good understanding of the main tephritid threats, of which *Bactrocera* species from across the Torres Strait (northern Australia) are of most concern. Effective tools for tephritid detection and early warning surveillance at points of entry are in place at ports and in horticultural areas Australia-wide and provide the basis for initiating biosecurity responses in the event of an incursion. Area-wide control measures used in successful eradication attempts globally are available for use in Australia. However, a specific tephritid emergency response plan identifying suitable response measures and control options for species of concern is not yet available. We have identified that Australia has the policies and management tools available to respond to an exotic tephritid incursion, but the speed at which this could be accomplished would be greatly improved by the development of species-specific emergency response plans.

## 1. Introduction

Tephritid fruit fly species are a serious threat to global food production. Fruit fly infestations cause significant economic losses, reduce commodity quality, and disrupt trade, which in turn affects livelihoods, food security and market access [1,2]. To date, Australia remains largely free of economically damaging exotic fruit fly species, such as the Oriental fruit fly (OFF; *Bactrocera dorsalis* (Hendel)). This freedom from most exotic tephritids means that Australian horticultural commodities can maintain premium prices and Australian horticultural industries are worth ~AUD 15 billion annually [3]. If an incursion by exotic fruit flies were to occur, even for a short time, market access for export produce would be challenged [4] and this would result in significant economic losses [5]. As such, it is vital that Australia has good biosecurity and management strategies in place to monitor for and respond to fruit fly incursions as they arise.

At present there are five exotic fruit fly species that have been identified as biosecurity priorities for Australian horticulture: *Anastrepha ludens* (Loew) (Mexican fruit fly), *Bactrocera carambolae* (Drew & Hancock) (carambola fruit fly), *Bactrocera dorsalis* (Hendel) (Oriental fruit fly (OFF)) (Note that *Bactrocera dorsalis* is considered the senior synonym of *Bactrocera papayae* Drew & Hancock, *Bactrocera invadens* Drew, Tsuruta & White, and *Bactrocera philippinesis* Drew & Hancock, with *Bactrocera carambolae* Drew & Hancock considered a separate species included in the *B. dorsalis* species complex [6].), *Bactrocera trivialis* (Drew) (New Guinea fruit fly) and *Zeugodacus cucurbitae* (Coquillett) (melon fly) (see Table 1) [7]. These species were identified during the development of the National Priority Plant Pests list by the Australian Government [7]. To be considered a priority pest, exotic species must be injurious to plants/plant products, have the potential to cause significant negative effects on the economy, environment or community, and have the potential to enter, establish and spread within Australia [7].

International horticultural trade and tourism from Australia’s nearest neighbours (predominantly countries in Asia) are the most probable pathways for an incursion by these tephritid species [8,9], although wind-assisted dispersal across the Torres Strait is also significant [10,11]. Except for the Mexican fruit fly [12,13], all the above species are present in Asia. *Bactrocera* species (particularly OFF) are of most concern to Australia, as species in this genus have a long history of invasion and establishment globally (e.g., [14]). OFF, regarded as the most damaging and aggressive fruit fly [15], has the highest incursion risk due to its presence throughout Asia [16], invasion and establishment in Indonesia [17] and Papua New Guinea [18], and because of the historical incursion and subsequent eradication of OFF (then *B. papayae*) in far north Queensland, Australia in the 1990′s [19].

Here, we aim to review Australia’s capacity for preventing and managing an incursion of exotic tephritid fruit flies. Observations of the history of incursions and subsequent eradication attempts, such as the incursion of OFF into Africa [14,20] or medfly (Mediterranean fruit fly, *Ceratitis capitata* (Wiedemann)) into almost every continent [21,22], can assist in developing best practice management strategies for control and damage mitigation. We provide a summary of national and international tephritid fruit fly incursion management practices, a summary of the history of economically damaging fruit flies in Australia and an outline of the potential of an incursion into Australia by exotic fruit fly. The aim of this study is not to provide an extensive review of exotic fruit fly management practices, as this has, to varying degrees, already been achieved (for examples see [13,23,24,25]. Rather, it is to examine if current Australian measures are adequate to manage an incursion by exotic fruit flies and, if not, to suggest modifications that will improve Australian management practices.

**Table 1:** The top five exotic tephritid fruit fly species of biosecurity concern to Australia, their distribution, hosts, ideal climate and known attractants.

Species	Distribution		Commercial Host/s	Climate	Lure
	Native	Invasive			
*Anastrepha ludens* (Loew) Mexican fruit fly	Mexico (other areas of North America possible)	United States (transient-under eradication in California and Texas) and Panama	Polyphagous. Major commercial hosts citrus and mango	Tropical and warm temperate	No para-pheromone lure known but captured in traps with ammonia based lures [26,27,28,29]
*Bactrocera carambolae* (Drew and Hancock) carambola fruit fly	ASIA including Malaysia, Thailand, Indonesia, Timor-Leste, Vietnam, Singapore, Myanmar, India, Cambodia	French Guyana, Guyana (eradicated 1994), Brazil, Suriname, Brunei, Bangladesh	Polyphagous >100 hosts [12]. Major commercial hosts carambola, mango, jackfruit, sapodilla, guava [30]	Tropical and sub-tropical [31]	Methyl eugenol [30]
*Bactrocera dorsalis* (Hendel) Oriental fruit fly (OFF)	Southeast Asia	>75 countries in Asia, North America, South America, Africa [32] and Oceania (Christmas Island, Papua New Guinea, Palau, Hawaii and Tahiti)	Polyphagous >300 cultivated fruit and vegetable host species. Most edible fruit is susceptible [12,33]. Some major hosts include mango, stone fruit, apple, citrus, cherry, carambola, cashew, banana.	Tropical and warm temperate	Methyl eugenol [7]
*Bactrocera trivialis* (Drew) New Guinea fruit fly	Papua New Guinea and Indonesia [34]		Polyphagous, recorded on 17 host plants [33]. Major commercial hosts chilli, grapefruit, mango, peach, guava [33,34]	Tropical	Cuelure [30]
*Zeugodacus cucurbitae* (Coquillett) melon fly	India	>40 countries including in Africa, Asia, Oceania (Papua New Guinea, Mariana Islands, Solomon Islands, Nauru, Kiribati, Guam, Hawaii [35]). Eradicated from Japan [36] North America (California)	Polyphagous but predominantly cucurbits. Recorded on 44 hosts [12,33] Main commercial hosts include watermelon, rockmelon, cucumber, pumpkin, bitter melon, edible *Luffa*, ivy gourd, wax gourd, bean and tomato.	Tropical and warm temperate	Cuelure group (melolure in Hawaii) [37]

Information summarised from CABI-Invasive species compendium www.cabi.org/isc/ (accessed on 15 January 2023) where correct and Plant Health Australia Fruit fly Identification website www.fruitflyidentification.org.au (accessed on 15 January 2023) and other identified sources, most up to date and correct information used.

## 2. Incursion and Environmental Suitability

### 2.1. Incursion and Dispersal Ability

The threat of incursion exists for all countries due to increasing global trade in fruit and vegetables [32,38,39]. Frequently, tephritid incursions follow a jump-diffusion model [40] where tourism (e.g., [9]) or trade provide the long-distance jump, followed by dispersal into the local environment, often with human assistance [11]. In Australia, local human movements appear responsible for Queensland fruit fly (Qfly, *Bactrocera tryoni* (Froggatt)) dispersal of up to several hundred kilometers (e.g., [41]). Internationally, human movements have contributed to the dispersal of medfly to most continents [21] and the spread of OFF through most of the Pacific Islands [42].

While trade and tourism represent the most likely incursion pathways for exotic fruit flies, wind is also thought to play a role, especially in Australia. In general, the influence of wind on fruit fly dispersal is not well understood and reports are contradictory. Some indicate wind does not redistribute tephritids across land (e.g., [43]), while others provide evidence for downwind dispersal [10,44]. Mark and recapture studies show that most fruit fly species do not move far from their release sites, being limited to distances of 2–4 km [43,45] although dispersal ability appears to be largely species-specific. There are a few instances where fruit flies, particularly *Bactrocera* species, have dispersed large distances. OFF and Qfly have been found at distances of 50 km and 94 km, respectively, from their release sites [43,46], although most reports indicate dispersal distances of less than 1 km. Conversely, other species such as melon fly and Mexican fruit fly do not disperse aerially over significant distances. Mark and recapture studies on the melon fly have demonstrated a dispersal distance of between 50 and 800 m from release sites [47,48], while Mexican fruit flies dispersed up to 9 km, but typically traveled less than 250 m [49].

For Australia, the influence of winds on dispersal may be more important across waterways, such as the Torres Strait [10,11]. There is strong evidence that prevailing wind patterns during the Australian northern wet season (November to April) facilitate the movement of exotic tephritids from southern areas of the Papua New Guinea (PNG) mainland onto the Torres Strait Islands [18]. The closest islands, Boigu and Saibai, lie less than 6 km from PNG, with the rest of the islands spread out over 150 km between Cape York on the Australian mainland and PNG. Trapping of fruit flies conducted as part of Australia’s surveillance network on the Torres Straight Islands frequently intercepts OFF, melon fly and New Guinea fruit fly [11,18]. It is possible that wind-assisted dispersal of exotic flies to the southerly Torres Strait Islands could lead to an incursion on the Australian mainland.

### 2.2. Climate, Fruit Fly Ecology, Hosts, and Incursions

Climate and host availability are the two most important factors governing the establishment and spread of fruit fly species during an incursion event. Understanding the response of fruit fly species to various climate scenarios in terms of their adaptability can allow for predictions of how incursions will develop for different species. For example, OFF has a reduced capacity (lower trait plasticity) to adapt to increasing temperatures when compared to medfly [50]. This difference in trait plasticity may indicate that high temperatures will favour medflies in some locations, even though thermal tolerance to high temperatures itself is greater in OFF (e.g., medfly [51]; OFF [2]) (see Table 2 for examples). These differences in trait characteristics have applications in models attempting to forecast distributions for exotic fruit fly species, such as CLIMEX [4,52], GARP [53] or MaxEnt [54,55]. These models can be used to predict climate-dependent limitations for survival and population growth, allowing incursion surveillance measures to be focused on regions with appropriate conditions for fly population establishment.

Models, such as those previously mentioned, suggest that with increasing global temperatures tropical areas are likely to become less suitable for many fruit fly species, as temperatures will exceed thermal limits for survival. Conversely, temperate areas will become more suitable due to a reduction in unfavourable winter conditions (e.g., extreme frost events) allowing for overwintering by adults [16,56] and consequently population establishment. Within Australia, most models of climate suitability for native tephritid fruit fly species (especially those of economic importance) indicate future temperature conditions will facilitate range expansion from Queensland and northern areas of NSW into southern NSW and Victoria along the eastern seaboard and to higher elevations along the Great Dividing Range [54,57,58]. Northern Queensland is predicted to become less acceptable to many local tephritids, but potentially more acceptable to the establishment of exotic species, particularly OFF (e.g., [53,54]).

**Table 2:** Examples of species traits that can be used in models predicting fruit fly establishment likelihood and distribution.

Trait		Bactrocera dorsalis * (Hendel) Oriental Fruit Fly (OFF)	*Bactrocera tryoni* (Froggatt) Queensland Fruit Fly (Qfly)	*Ceratitis capitata* (Wiedemann) Mediterranean Fruit Fly (medfly)
Critical thermal limits	min	7.3 °C (larvae) [2] 9.1 °C (adults) [2]		6 °C (adults) [51,59]
	max	45.23 °C (larvae) [2] 46.16 °C (adults) [2]		42 °C (adults) [51,59]
Lethal limits	lower	−8 °C (larvae) [2] −6 to −6.5 (adults) [2,50]		−7 to −3 °C all life stages [60]
	upper	42 to 45 °C (adults) [2,50]	38–40 °C (all life stages) [61]	37 °C all life stages [60]
Developmental temperature thresholds	lower	8.8 °C,11.8 °C, 12.1 °C, 12 °C (eggs) [62,63,64,65] 5.6 °C, 9.4 °C, 10.5 °C (larvae) [62,63,64] 8.7 °C, 9.3 °C, 10.9 °C (pupae) [62,63,64]	12 °C (all life stages) [65]	10 to 11 °C (egg) [63,66] 5 °C, 10.2 °C (larvae) [63,66,67] 9 °C (pupae) [63]
	upper	30 to 35 °C (all life stages) [62]	37 °C (eggs) [61,65] 35 °C (larvae) [65] 34 °C (pupae) [65]	35 °C (larvae) [2]
	optimum	24–30 °C (all life stages) [62,68]	25–30 °C (all life stages) [61]	24–27 °C (all life stages) [62,68]

* Includes data from *Bactrocera dorsalis* synonymy.

Despite forecasts that an area is or will be climatically suitable for range expansion by native or exotic tephritids, establishment can be limited by host availability or by interspecific competition for hosts from local fruit fly species. In an Australian context host availability alone might not be a limiting factor for establishment as the exotic tephritids in our study are polyphagous (Table 1; Table 3), i.e., OFF affects more than 250 fruits and vegetables [32], and the most likely entry points for an incursion are airports and seaports which are typically in or near capital cities and which are generally surrounded by peri-urban and food production areas with a wide variety of hosts [69].

Competition for hosts between native and exotic fruit fly species has the potential to curb the establishment of exotic species. Research [70,71] suggests that the ability of an invading species to overwhelm resident species is partly based on their reproductive capacity, or the number of adult flies produced per kg of fruit (termed the *host reproduction number* (HRN) [72]). OFF has frequently become the dominant tephritid species after introduction to a new country or region [42], outcompeting resident fruit fly species [2,71,73]. OFF has a high HRN in many hosts (see Table 4 for examples) and a competitive advantage over other species, which is largely due to its ability to infest unripe fruit (e.g., [74,75]). Laying eggs in unripe fruit means that OFF can often claim resources before other species [71,76]. HRNs are available for other fruit fly species (see [72] for medfly HRN), but information is limited. For Australian incursion management, a comparison of Qfly (the most economically damaging and widespread endemic fruit fly species) and exotic fruit fly HRN values would be beneficial to increase the accuracy of determining the invasion and establishment potential of exotic fruit fly taxa [77]. A caveat to this is that in certain locations such as northern Australia, especially in island habitats, host type may be very limited. In these areas, the benefits of using HRN for optimising fruit fly management may also be limited, especially in areas where hosts are similar across fruit fly species.

**Table 3:** Australian pest species of tephritid fruit fly, their distribution, hosts, preferred climate and attractant (lure type). *C. capitata* and *B. frauenfeldi* are the only non-native species. Note *B. aquilonis* and *B. neohumeralis* are sibling species of Qfly [69,78] and *B. aquilonis* is genetically indistinct from Qfly [79].

Species	Distribution		Host/s	Climate †	Attractant
	Native	Invasive			
*Bactrocera aquilonis* (May) Northern Territory fruit fly	Northern areas of Western Australia and the Northern Territory		Polyphagus [80], major host billy-goat plum (*Terminalia ferdinandiana*). Major commercial host bitter gourd and guava	Tropical	Cuelure
*Bactrocera bryoniae* (Tryon)	West Papua, Papua New Guinea, Queensland, Northern Territory, New South Wales and northern Western Australia [81], and Torres Strait Islands [35]		Polyphagous, recorded on 9 hosts [80], main host chilli	Tropical, sub-tropical	Cuelure group (melolure in Queensland [82]) and Wilson’s lure [34]
*Bactrocera frauenfeldi* (Schiner) mango fruit fly	Federated States of Micronesia, Kiribati, Marshall Islands, Palau, Nauru, Solomon Islands, Moluccas, Papua New Guinea.	Invasive to Queensland and Torres Strait [83]	Polyphagous, 109 known hosts. Commercial hosts mango, banana, citrus, carambola, guava, papaya, edible *Syzygium*, star apple, sapodilla and abiu [33,80]	Tropical	Cuelure group (melolure [82])
*Bactrocera jarvisi* (Tryon) Jarvis’ fruit fly	Queensland and northern tropics [84] including Western Australia, Northern Territory, Torres Strait Islands and NSW south to Sydney [80]. West Papua and Papua New Guinea [33]		Polyphagous. Recorded on 84 hosts. Main host cockatoo apple (*Planchonia careya*). Commercial hosts include mango, guava, papaya, persimmon, soursop, avocado, banana, pomegranate, apple, peach, pear, coffee, citrus and edible *Syzygium* [80]	Tropical, sub-tropical, warm temperate	Zingerone [85] and weakly to cuelure [84]
*Bactrocera kraussi* (Hardy)	Torres Strait Islands and northeast Queensland south to Townsville [80]		Polyphagous. Recorded on 106 hosts. Commercial hosts include mango, banana, grumichama, feijoa, carambola, peach, citrus and tamarind [80]	Tropical	Isoeugenol and weakly to cuelure [82]
*Bactrocera musae* (Tryon) banana fruit fly	Torres Strait Islands and northeast Queensland south to Townsville [80], Papua New Guinea and associated islands [34], West Papua [35]		Polyphagous. Recorded on 16 hosts. Primary economic host banana, occasional hosts papaya and guava [80]	Tropical	Methyl eugenol
*Bactrocera neohumeralis* (Hardy) lesser Queensland fruit fly	Eastern seaboard [69,78] south to Coffs Harbour, Papua New Guinea		Polyphagous recorded on 160 hosts. Commercial hosts include mango, custard apple, rollinia, date palm, persimmon, mulberry, banana, carambola, passionfruit, loquat, apple, plum, peach, pear, citrus, coffee, star apple, sapodilla, abiu, capsicum and tomato [34,80]	Tropical, sub-tropical	Cuelure group [34]
*Bactrocera tryoni* (Froggatt) Queensland fruit fly (Qfly)	Queensland, New South Wales and Victoria [86], Northern Territory [87].	Invasive to New Caledonia, French Polynesia, Pitcairn Island [34]	Polyphagous recorded on over 200 hosts. Major commercial crops include mango, custard apple, papaya, carambola, passionfruit, loquat, apple, peach, coffee, star apple, sapodilla, capsicum, chilli and tomato [34,80]	Tropical, sub-tropical, warm temperate	Cuelure group [82]
*Ceratitis capitata* (Wiedemann) Mediteranean fruit fly (medfly)	African sub-Saharan countries.	Invasive to >60 countries- Middle East, Europe, Egypt, Americas, and Australia (Western Australia and incursions into South Australia [86]).	Polyphagous (any type of fleshy fruit-location dependant), major commercial hosts cherimoya, bell pepper, citrus, coffee, fig, apple, stone fruit, Japanese plum, guava, cocoa.	Mediterranean and warm temperate (elevated tropical regions)	Trimedlure/capilure, EGO lure and terpinyl acetate [87], enriched ginger oil (EGO) [88]
*Zeugodacus cucumis* (French) cucumber fruit fly	Northern Territory, Queensland, Torres Strait Islands, northeast New South Wales [80]		Polyphagous recorded on 40 hosts. Major commercial hosts include papaya, cucumber, pumpkin, squash, zucchini, guada bean, passionfruit and tomato	Tropical, sub-tropical	Not attracted to male lures, cucumber volatile blend attractive to both sexes [89,90]

Data obtained from the Australian Handbook for the Identification of Australia fruit fly [30] and associated website fruitflyidentification.org.au (accessed on 15 January 2023) unless otherwise stated. † Based on presence data.

**Table 4:** Examples of very good host plants for *Bactrocera dorsalis* and associated host reproduction number (HRN) from different regions. HRN is calculated as the number of adults who emerged per 1 kg of fruit and very good hosts have a HRN value exceeding 100 [91]. HRN is for affected fruit in the field.

Host	Africa	Asia and the Pacific
*Annona muricata*—Soursop [92]	454	
*Carica papaya*—Pawpaw [93]		142
*Chrysobalanus icaco*—Icaco [94]	125	
*Diospyros blancoi*—Velvet apple [94]	140	
*Eriobotrya japonica*—Loquat [92]	325	
*Mangifera indica*—Mango [92]	505	
*Musa* sp.—Banana [12,95]	130	247
*Nephelium lappaceum*—Rambutan [95]		370
*Pometia pinnata*—Pacific lychee [95]		195
*Psidium guajava*—Guava [42,96]	156	247
*Sclerocarya birrea*—Marula [12]	238	
*Syzygium jambos*—Jambos, Rose-apple [94,95]	141	225
*Terminalia catappa*—Tropical almond [12]	653	
*Vaccinium corymbosum*—Blueberry [95]		1228

## 3. Management Strategies for Tephritid Fruit Fly Incursions

### 3.1. Emergency Response Plans

Effective control of exotic fruit fly incursions starts with an emergency response plan, which many countries have in place to guide their response operations. Plans typically involve identification, trapping (to delimit the incursion), host sampling surveys to identify the incursion zone, the establishment of a quarantine zone (usually with several levels or buffer zones including an exclusion zone) and then eradication (e.g., [97,98]). Australia has a generic emergency response plan, the *Australian Emergency Plant Pest Response Plan* or *PLANTPLAN* [99]. *PLANTPLAN* sets out general guidelines to follow in the event of an incursion by a plant pest. In summary, this includes identification of the pest (initial and confirmatory morphological or molecular diagnostics), determining the geographic extent of the incident, establishing quarantine zones (restricted areas, control areas and pest-free areas), establishing movement restrictions (for vehicles/machinery/produce) and finally control strategies (see Table 5 for fruit fly control examples). To date, mainland Australia does not have a generic or species-specific emergency response plan for exotic fruit fly species. There is, however, a detailed response plan (not an emergency plan because it manages ongoing seasonal incursions) for exotic fruit flies called the *National Exotic Fruit Fly in Torres Strait Eradication Program: Response Plan 2021–2026*. This plan describes the response activity required for detection and control of OFF, melon fly and New Guinea fruit fly and is based upon historical detections and proven eradication activities refined over the last 25 years in this area. While the implementation of this plan is useful to prevent incursions into Australia and acts as an early warning system, it does not cover the response to any mainland incursions [18], and as such the addition of a specific emergency response plan (as part of a National Action Plan) for exotic fruit fly species is required for mainland Australia.

### 3.2. Border Control and Surveillance

International border controls, entry restrictions, policies and treatment protocols are employed globally by countries to stop the entry of infested produce (fruit and vegetables) carried by travellers or in commercial consignments (e.g., [110,111]). Commercial fruit consignments are usually required to be treated using a recognised disinfestation technique to eliminate any live insects within the fruit. There are three main disinfestation techniques: irradiation [112], temperature treatments [113,114] and fumigation (e.g., methyl bromide) [115]. Methods used for disinfestation typically vary between crops due to associated damage, quality loss or acceptance of treatments by trading partners. Irradiation is highly effective against many tephritids with an internationally accepted dose of 150 Gy [112,116]. Temperature disinfestation is a non-polluting strategy but is time-consuming, may affect quality and typically has a large energy footprint. Methyl bromide is an ozone-depleting substance and is scheduled for phase-out under the Montreal Protocol. Despite quarantine treatments currently having a critical use exemption, the use of methyl bromide is likely to become increasingly restricted [115], and many countries including the European Union have banned its use for quarantine purposes on imported products.

Interceptions by border control are very successful in preventing incursions. Australia has some of the strictest biosecurity border controls in the world. The *Department of Agriculture, Fisheries and Forestry* (DAFF) and *Australian Border Force* manage pre-border checks of produce which involve declaration, manual inspection, X-ray inspection, inspection by detection dogs and destruction or treatment of seized goods [117]. In Australia, between 2012 and 2018, 1.9 million items of biosecurity concern were seized at airports. Between 2014 and 2017 there were over 37,000 border interceptions of pests (most of which were insects). Of these interceptions, most pests (45%) were brought into Australia by air cargo followed by sea cargo and containers (35%), the remainder a combination of interceptions from international air passengers and mail. Less than 1% of interceptions were for high-priority plant pests [117]. Border controls are vital in preventing incursions as propagule pressure (the initial population size) is linked to establishment success—the smaller the propagule the less likely a population will establish [77].

Should fruit flies bypass pre-border controls, targeted surveillance is used to detect flies or provide evidence of early establishment. Surveillance varies widely between countries. Australia, New Zealand and the USA (California and Florida) all have extensive surveillance networks in place (see [101] for a review; [118] for a map of the surveillance network in Australia). Typically, surveillance networks consist of grids interspersed with traps (e.g., McPhail, Lynfield, or Steiner traps) laced with a lure and an insecticide (e.g., malathion, fipronil, spinosad). Lures can be species or genus-specific, such as para-pheromone lures designed to attract males only or ammonia-based lures for non-lure-responsive species, which capture both sexes. Most if not all fruit flies are attracted to ammonia-based lures, and baits of ammonia laced with insecticides are commonly used for control or eradication of fruit fly populations (see Section 3.3 *Eradication and control*). However, para-pheromone lures are typically much more effective in terms of detecting target species for surveillance purposes. Ammonia-based lures are an old technology used before para-pheromones became available and are typically used where no para-pheromone lure has been developed for a particular species. The para-pheromone lures methyl eugenol, cuelure and trimedlure/capilure are widely used to attract OFF, Qfly and medfly males, respectively. In contrast, the Mexican fruit fly has no known para-pheromone lure, but ammonium acetate, hydrolysed protein, torula yeast and putrescine-based lures work as attractants [26,27,28,29]. Table 1 and Table 3 list the lures used for the species mentioned in this review.

Fruit fly trap and lure selection is influenced by the target fly species and by environmental conditions, leading to different trap/lure combinations being used in different countries or regions. USA surveillance programs (in California, Florida and Texas) use Jackson traps in combination with trimedlure and BioLure^®^ (ammonia-based) for medfly, BioLure^®^ for *Anastrepa* spp., cuelure and methyl eugenol for *Bactrocera* spp., and McPhail traps baited with torula yeast to monitor Mexican fruit fly [119]. The *New Zealand Fruit Fly Detection Program* uses Lynfield traps baited with cuelure to attract Qfly and melon fly, methyl eugenol to attract OFF, trimedlure/capilure to attract medfly, and ammonia-based lures as a general lure for species not attracted to para-pheromones [120,121] (see below for details of Australian trap/lure combinations).

Typically, surveillance networks employ grids of traps at densities of between one and eight traps per km^2^ at major ports of entry and in high-risk areas (i.e., urban areas and horticultural zones), depending on designated risk [101,122]. The effectiveness of a trapping grid in detecting incursions is dependent on the sensitivity of the grid, which in turn is determined by the type and combination of lures used (some combinations of lures can reduce efficacy (e.g., [123]), grid size, trap density, monitoring frequency, geography and climate [124]. Additionally, the use of any particular trap/lure combination in lure-based eradication designed for one fruit fly species may lead to an increase in the likelihood of the establishment of other species. The suppression of resident fruit fly populations may reduce competition for invading species [125], and the traps and lures in place may not be suitable for the control of the new invading species [126]. Trapping guidelines have been issued by the *Food and Agriculture Organisation* and *International Atomic Energy Agency* [124] and stipulate trap density for effective monitoring, control/suppression and eradication.

The fruit fly surveillance network in Australia is complex, as it is largely funded by the federal government but run independently by the Australian states and territories. The federal program is responsible for international border control and runs the *National Plant Health Surveillance Program* (NPHSP) (previously the *Australian National Exotic Fruit Fly Surveillance Program*), and the *Northern Australia Quarantine Strategy* (NAQS) (see [86,101]). The NPHSP was established after the 1995 incursion of OFF in Cairns and consists of a network of 4800 traps equally baited with a malathion toxicant and cuelure, capilure or methyl eugenol lures set up at one trap per 25 km^2^ within 20 km of major ports of entry around Australia [101] (see [127] for a location map).

NAQS became operational in 1989 and remains the key Australian program today to monitor incursions from non-regulated pathways into northern Australia [101]. The NAQS fruit fly monitoring network currently comprises 125 permanent traps spread out over 20 islands at 46 sites, as well as across 13 sites in the northern tip of Cape York Peninsula, Queensland [18,127]. Additional response traps are added in the event of an incursion.

Each state and territory in Australia additionally runs its own fruit fly surveillance programs, mostly to monitor the spread of existing but regionally restricted fruit fly populations (i.e., Qfly and medfly). These programs act as an early warning system for incursions and also demonstrate the absence of fruit fly species for international trade purposes [69]. In combination, the trapping surveillance networks of the Australian states and territories consist of more than 25,000 traps baited with a combination of methyl eugenol, cuelure and capilure, set up at a density of 6 traps per km^2^ in high-risk urban areas and at 1 trap per km^2^ in horticultural zones [69,100,115].

Surveillance trap architecture varies between locations due to local environmental conditions, predominantly rainfall frequency. The Lynfield trap is used throughout NSW [69], in most southern regions [128] and in many parts of southern and central Queensland [129]. In northern Australia, Lynfield traps are suboptimal due to heavy monsoon rain and Paton and Steiner traps are used instead [11]. Recently, Biotraps^®^ were found to be equivalent to Lynfield traps in New Zealand and southern Australia in terms of efficacy [130] and cone traps were also found to be equivalent to Lynfield traps [131]. These trap designs may replace Lynfield traps as their availability declines [132]. Based on national guidelines, most states inspect their fruit fly traps fortnightly. Traps for the Torres Strait grid are checked monthly during the “dry” season (July to December) and fortnightly during the “wet” season (January to June) [18]. Data from programs (excluding NAQS trapping data) are sent to the federal government agency, the DAFF to include in a restricted-access national database called AUSPestCheck^®^ run by *Plant Health Australia* (PHA), the national coordinator of the government-industry partnership for plant biosecurity in Australia.

### 3.3. Eradication and Control

According to the *Global Eradication and Response Database* (GERDA), [133] there have been approximately 189 incursion responses and eradication attempts for the exotic fruit flies mentioned in this study; nine for Mexican fruit fly in Mexico and the USA, eight for melon fly in the USA and Japan, approximately 55 for OFF (including synonyms) in USA, Japan, Australia, New Zealand and French Polynesia and 117 for medfly in New Zealand, USA, Australia and South America (for a summary see Table 6, for full information on successful eradications see http://b3.net.nz/gerda accessed on 15 March 2023). By summarising the methods used, we found that a combination of management technologies including cultural control (e.g., wrapping fruit, early harvesting, crop sanitation, soil raking), establishment of quarantine zones, the male annihilation technique (MAT), the bait application technique (BAT), the sterile insect technique (SIT) and biological control agents were used in an area-wide manner in successful eradication programs (see below and Table 5 for definitions). The most common eradication methods included various combinations of MAT, BAT and SIT.

MAT is a low-cost management strategy designed to attract and kill males so that mating is severely compromised [104]. MAT is used predominantly in orchards and urban control of fruit flies [19]. MAT uses para-pheromone lures (e.g., methyl eugenol or cuelure) in combination with an insecticide (e.g., malathion). The lure and insecticide are impregnated into caneite (compressed fibreboard), cordelitos (cotton wicks), coconut husks [18], compressed cardboard [18,137] or plastics [103]. The choice of material to be impregnated depends on rainfall, geography and local availability. Areas of high rainfall require more frequent replacement of MAT assemblies. Where access is difficult, MAT assemblies using cordelitos can be dropped from aircraft and remain active for several months. The Torres Strait Islands program in Australia uses caneite blocks impregnated with malathion and methyl eugenol or cuelure [18] for control of exotic fruit flies. Likewise, caneite blocks impregnated with malathion and methyl eugenol formed a very effective part of the Cairns eradication of OFF in the 1990s [19,102].

BAT or protein bait spays are another key component of fruit fly control, where a toxicant and attractant are applied to the foliage of host crops, usually only along the borders of the crop or in targeted areas. The toxicants and attractants used vary between countries, but toxicants may include malathion [105], spinosad [106,138] or fipronil [107]. Both male and female flies are attracted to protein sources emitting ammonia, so often the attractant used is a protein-based material. Either protein hydrolysate [103] or protein autolysate [139] is typically used, although protein hydrolysate may be phytotoxic due to its high acidity. In Australia, the type of protein used in BAT to control Qfly was changed from protein hydrolysate to protein autolysate for exactly this reason [108]. Malathion is the most frequently used BAT toxicant in Australia [11,18,127,140]. However, as the ability to use pesticides becomes more restricted by the *Australian Pesticide and Veterinary Medicines Authority* (APVMA) due to environmental and human health concerns, Australian chemical control of fruit fly is moving towards toxicants such as spinosad (a toxicant derived from the actinobacterium *Saccharopolyspora spinosa* Mertz and Yao (Pseudonocardiales: Pseudonocardiaceae) that is non-toxic to mammals but highly effective against arthropods (e.g., [18], for a review of chemical use against fruit fly in Australia see [141]).

SIT is the process of mass-rearing and releasing large numbers of sterile flies (preferably males only) into a wild population, the intended outcome being that wild females will mate with sterile males and then not produce fertile eggs, which in turn reduces the population [109]. SIT is an expensive and complex control method, but also a more environmentally sensitive option for fruit fly control. It requires specialised facilities and proven rearing, transportation and release techniques to minimise losses of sterile flies. This method of control has been used successfully for eradication as well as in reducing population size [136,142]. Australia has used SIT to suppress outbreaks of local fruit fly species since the 1960s [143,144] and currently has two SIT facilities available, one in South Australia and one in Western Australia, that produce sterile Qfly and medfly adults.

### 3.4. Economic Costs of Fruit Fly Incursion Management

The costs of fruit fly incursion management including preventative measures such as surveillance, eradication costs and loss of profits vary considerably but tend to range from millions to billions of dollars, depending on the location and the extent of the incursion. It is typically much less costly to invest in good incursion prevention measures than to initiate an eradication response. In Australia, an exotic fruit fly incursion would result in production disruptions, potential loss of domestic and international markets and income losses, together estimated at between AUD 269 million and AUD 2.1 billion, depending on the area affected and the success of the eradication attempt [18]. The cost of surveillance in mainland Australia in 2002 was estimated to be between AUD 1.3 and AUD 7 million per year [145] and the total cost of the NAQS surveillance program from 2021 to 2026 is estimated at AUD 4 million [18]. The eradication of OFF from Australia in the 1990s cost an estimated AUD 34 million [19], with a further AUD 100 million in costs incurred through increased production expenses, reduced productivity, and market access closures [18].

## 4. Fruit Fly in Australia

### 4.1. History of Economically Important Fruit Flies in Australia

Of the 301 tephritid species native to Australia [146] only eight are considered problematic to Australian horticultural industries [30] (Table 3). The most widespread and damaging of these tephritids is Qfly [140]. Qfly is thought to be native to Queensland and the north New South Wales coast. Historically, southern Australia was climatically suitable for Qfly establishment, however, range extension did not occur until after European settlement [40] brought the unregulated domestic trade of fruit and vegetables [86]. Qfly was first reported in NSW in 1819, but it was not considered a pest in NSW until 1852, with the first major outbreak occurring in 1884 [34]. Qfly is also present in the Northern Territory [140] and has been found periodically in (and eradicated from) South Australia, Western Australia [128,140] and Tasmania [147].

There have been two noteworthy incursions of exotic fruit flies into Australia. The first major incursion was by medfly. Medfly is considered the second most economically damaging fruit fly in Australia, after Qfly. Medfly was first introduced into Western Australia in 1896 and New South Wales in 1898 [148] and had spread to Victoria by 1909 [149]. It disappeared from the eastern states by the 1950s, probably due to a combination of control measures (most likely fruit destruction and soil drenches) and competition from Qfly [140]. Since that time there have been sporadic and swiftly eradicated outbreaks of medfly in South Australia (mostly around Adelaide) and in the Northern Territory [133,149]. In general, for national management and international trade purposes, Medfly is considered restricted to Western Australia, while Qfly is restricted to eastern areas of Australia (Northern Territory, Queensland, NSW, Victoria) and South Australia and Tasmania are free from both pests.

In the second major incursion, OFF (then *B. papayae*) was detected in papaya (*Carica papaya*) near Cairns in northern Queensland in October 1995, and again (as *B. philippinensis*) in the Northern Territory in November 1997 [19]. A national eradication response was mounted by state and federal government agencies and by 1999 OFF was eradicated from mainland Australia [19,146]. To date, mainland Australia remains free from OFF, however as mentioned previously interceptions and eradication of OFF (and other species) still occur yearly in the Torres Strait Islands [18,118].

### 4.2. National Fruit Fly Programs and Policy

The *Australian Plant Health Committee* (PHC) is the peak government plant biosecurity policy and decision-making forum. It provides strategic policy, technical and regulatory advice and national leadership on all plant biosecurity matters to protect Australia’s plant health and economic benefits derived from fruit and vegetable exports. In 2011, at the request of the *National Biosecurity Committee* (NBC), PHC assumed responsibility for managing policy for endemic fruit flies and the *Australian Fruit Fly Technical Advisory Subcommittee* (AFFTAC) was commissioned to develop *Australia’s National Fruit Fly Management Protocol* (ANFFMP). AFFTAC provides the necessary technical support for a nationally coordinated approach to surveillance and management of fruit flies, in line with Australia’s international import and export market access conditions and policies.

To manage ongoing fruit fly concerns in Australia a *National Fruit Fly Council* (NFFC) jointly funded by government and industry through their Research and Development Corporation (Hort Innovation) was established in 2015. The NFFC connects growers and fruit fly management groups across states and territories to control fruit flies in Australia on a national scale. The NFFC has produced a *National Fruit Fly Strategy* (NFFS) [150] to provide a framework for the cost-effective and coordinated management of fruit flies in Australia. It is designed to maintain Australia’s freedom from exotic fruit fly species, minimise the incidence and spread of local fruit fly species, support market access and facilitate a national approach to fruit fly management and research [150]. Other relevant plant protection organisations, such as *Plant Health Australia* (PHA), are responsible for developing and publishing important documents on the identification of native species of fruit fly (i.e., the Australian Handbook for Identification of Fruit Flies) and factsheets on the control measures that can be used against them.

In the event of an incursion of fruit flies Australia relies on a committee of experts called the *Consultative Committee for Emergency Plant Pests* (CCEPP) [151]. The CCEPP was developed as a response to the 1995 incursion of OFF to support a national response to fruit fly incursions. Before this time fruit fly management by the Australian states and territories was managed independently [19]. The CCEPP is designed to make recommendations for incursion management and oversees the preparation of a response plan (post-incursion) based on the general emergency response plan for plant pests (*PLANTPLAN,* [99]. This strategy is informed by a *National Management Group* (NMG) of Australian and international experts who will guide the incursion response based on the latest scientific information. The NMG usually stays engaged with the response and assists in the declaration of successful eradication if this is achieved.

### 4.3. Fruit Fly Incursion in Australia

Exact invasion pathways often remain unknown when an incursion occurs (e.g., [19]). As mentioned previously tourists, visitors and returning Australians from the Torres Strait Islands and Asia into northern Queensland are considered the most likely sources of an incursion [118]. Darwin (Northern Territory) and Cairns (Queensland) have international airports that service both tourism and airfreight and in addition to various potential incursion pathways, northern Australia also has excellent environmental conditions for fruit fly establishment and spread. Northern Australia has a tropical climate that is preferred by most exotic tephritids (see Table 1 and Table 3), and a wide range of hostplants are readily available. This area is particularly suitable for OFF establishment and additionally, the proximity to the Torres Strait Islands offers a potential stepping stone for OFF from PNG [18].

Surveillance traps are in place across Australia and contain fly-specific lures suitable for most species of concern, such as methyl eugenol which is attractive to OFF [11,115]. Mainland Australia seems well placed to detect any incursion, provided the surveillance trapping grid is maintained. Part of a good surveillance system is predicting where entry is most likely to occur to target priority areas for surveillance. There are several older models available that predict environmental suitability for OFF in Australia (e.g., [53,54]), however, models of environmental suitability are lacking for other species of concern and these types of models require frequent updating with the latest data to improve accuracy. Predictions for OFF indicate that the northern coastline of Australia is currently the most suitable [53], however, most of the eastern seaboard of Australia will be suitable for OFF establishment within the next 50 years [54,152].

Host availability is a key factor for fruit fly colonisation and establishment [153,154]. In most climatically suitable areas of Australia, there are no host limitations. However, there may be competition for hosts for invading fruit fly species. Qfly is established in most Australian horticultural areas and there are at least seven other endemic pest tephritids in northern Australia (Table 3; [55]), all of which currently compete for host resources [125]. An indication of the likelihood of establishment success might be gained by comparing the HRN of species of concern (e.g., OFF) with the HRN of native Australian tephritids [91]. Comparisons of OFF and Qfly on the more common and preferred host species such as mango would be of particular value. Unfortunately, HRN values for most fruit fly species present in Australia (other than Qfly and Medfly) are yet to be determined even for major hosts, and the competitive potential of these species cannot be easily assessed. This is important as it is thought that competition from native tephritid species masked the detection of the 1997 incursion of OFF in Cairns, which is estimated to have occurred 2.5 years prior to discovery [19].

Limiting factors to the spread of OFF in Australia are likely to be related to water availability, host availability and temperature. Areas of lower humidity cause moisture stress in tephritids and this would hypothetically restrict most exotic fruit flies to coastal areas of Australia since inland Australia is mostly arid. Lower temperatures that occur in southern Australia (which currently has a more temperate climate) would typically cause cold stress [154], with temperatures in large areas of southern Australia falling below the minimum requirements for development and survival (see Table 2). However, the availability of irrigation in the drier areas of southern Australia, both in towns and on farming land, climate change, the heat island effect of urban areas and host availability in backyard gardens may aid in survival and establishment in areas where natural conditions would have previously made this unlikely [152,153,154]. Anthropogenic environments (particularly in rural locations) need to be considered when attempting to model the establishment and spread of exotic fruit fly species in Australia. Models incorporating anthropogenically modified environments (e.g., [155]) show the establishment of fruit fly populations to be possible in areas where models incorporating only general climate data predict low or no establishment potential. Likewise, consideration of areas where the establishment is known to not occur, despite modelling predictions to the contrary, needs to occur. During the 1995 incursion OFF demonstrated an apparent inability to establish in natural rainforest habitats in far north Queensland, despite the large number of fruiting trees available in rainforest areas adjacent to horticultural crops. OFF establishment was restricted to areas of human habitation [19,156], which aided significantly in its successful eradication.

Australia has been successful in managing outbreaks of local fruit flies (Qfly and medfly) across state borders [115]. To control or locally eradicate Qfly and medfly, Australia uses various combinations of MAT, BAT [18], SIT [157], quarantine zones [19,140], surveillance, fruit removal and destruction [133], community awareness and engagement and, where necessary, financial penalties for transporting unauthorised produce into or out of quarantine zones [19]. These methods are all used in the control and eradication of exotic fruit fly species in global locations (Table 6) and could be used in the management of an incursion into Australia by exotic fruit fly species.

## 5. Conclusions

The high risk of a new incursion by OFF or by other exotic fruit fly species remains one of the primary drivers for continued targeted surveillance and quarantine measures at ports of entry into Australia [69,100]. Australia relies heavily on surveillance grids and border controls to prevent incursions of exotic tephritids. The maintenance of an effective surveillance and early warning system remains the best way to optimise Australia’s ongoing freedom from exotic fruit flies. The current surveillance system was established in the 1990s with reference to earlier environmental conditions, tephritid species and trapping methods. To be effective, the surveillance system in Australia needs to be utilising the latest and most effective lure types [158], best trap architecture, optimal grid spacing, appropriate pesticides [141,159], and trap locations based on detailed species distribution models and climate forecasts, with emphasis on busy ports of entry. A review of the national trapping grid was completed in 2018 by the DAFF, however, the results of this review, along with up-to-date information on the surveillance grid and its effectiveness, are not yet publicly available. Notwithstanding the above, we acknowledge that the current surveillance system seems to be effective, as there have been no serious incursions since the grid was established.

Specific areas of improvement other than those already mentioned, include a better understanding of the effect of interspecific competition between exotic and native species of fruit fly through the generation of additional HRN data. These data may help us better understand the establishment, detection and spread of exotic fruit fly species, as well as facilitate more closely targeted lure use. Lure type is particularly important for those species not attracted to the para-pheromone lures that are used in the Australian surveillance grid, such as Mexican fruit fly. Although Mexican fruit fly is not as high a biosecurity risk as OFF, Australia seems less prepared for Mexican fruit fly than for other priority species. This reflects the fact that whilst this species is listed as one of those of most concern to Australia, it is not present in Asia from where the greatest incursion risks are likely to originate. Additionally, the Mexican fruit fly is low on the ‘tephritid hierarchy’ [160] and is likely to have difficulty establishing in an environment saturated with *Bactrocera* spp. Surveillance for Mexican fruit fly (and other non-lure responsive species) could potentially be improved with the addition of ammonia-based lures that attract a wide range of fruit fly species to the Australian surveillance grid. This approach is being used by New Zealand in their fruit fly surveillance program [120,121].

Australia has a robust set of management strategies and policies available for the surveillance, containment, and eradication of local fruit fly species. To some degree, this has been extended to exotic fruit fly species. Australia-wide management protocols, strategies and plans for exotic fruit fly species, such as those discussed here, have benefited from the experience gained in implementing control measures directed towards preventing the internal spread of Qfly and medfly in Australia. Measures that have been used to successfully eradicate exotic tephritids (including OFF) globally also provide a sound basis for future incursion responses. National participation in management programs, the development of policy and collaboration by industry and government mean that Australia is reasonably well placed to deal with an incursion by exotic fruit fly. However, the lack of a specific emergency response plan means that the reaction to a post-border detection of exotic fruit flies will likely be delayed while research is conducted into the best management strategies for containment and eradication, potentially leading to a more severe and costly incursion outcome. A targeted emergency response plan would be relatively easy to develop and established management strategies for pest tephritids currently present in Australia would provide it with a strong foundation.

## Figures and Tables

**Table 5:** List of area-wide control options for fruit flies.

Control Strategy/Type	Description
Surveillance	Lure-based traps (para-pheromone or ammonia-based) used to monitor areas of concern (i.e., ports of entry, horticultural areas [100] for an incursion. Usually set up in a grid pattern [101].
Quarantine zones	Areas that restrict the movement of potentially infected fruit and vegetables to create “fly free” zones, this could be a country, an area of high horticultural value or an incursion area within a country [86].
Cultural control	The use of physical control techniques, such as crop sanitation and hygiene e.g., wrapping fruit, removal, and destruction of fallen fruits, digging up the ground to destroy pupae.
Cover sprays	Spraying pesticides directly onto affected crops.
Male annihilation technique (MAT)	Para-pheromone lures (e.g., methyl eugenol) and an insecticide (e.g., malathion) are impregnated into caneite (compressed fibreboard) [102], cordelitos (cotton wicks), coconut husks, compressed cardboard [18] or plastics [103] to lure and kill males so that mating is compromised [104].
Bait application technique (BAT) or protein bait sprays	An insecticide (malathion [105], spinosad [106], fipronil [107]) and protein source (protein hydrolysate [103] or protein autolysate [108]) is applied to the foliage of host crops, usually only along the borders of the crop or in targeted areas.
Sterile insect technique (SIT)	Mass-rearing and releasing millions of sterile flies into a wild population of flies, the intended outcome that females will mate with sterile males and not produce fertile eggs which in turn reduces the population [109].
Biological control agents (BCAs)	Natural control agents such as predators, parasitoids (wasps and flies), fungi, nematodes, bacteria and viruses to reduce populations [25].

**Table 6:** Successful eradication programs for the five tephritid fruit fly species of biosecurity concern to Australia and methods used in the eradication process.

Species	Location	Incursion/s	Eradication Program/s	Method/s of Eradication
*Anastrepha ludens* Mexican fruit fly	California, USA (multiple locations)	2000s	Multiple programs 1990–2009	BAT (spinosad), quarantine and movement control and SIT
	Mexico (multiple locations)	1980s	1992–1994	Natural enemies (parasitoids), BAT, host removal and destruction, quarantine and movement control, and SIT
	Texas, United States (multiple locations)	2008, 2011, 2013	2008–2013	BAT (spinosad or malathion), host removal or destruction, and SIT
*Bactocera carambolae* carambola fruit fly	Guyana	unknown	1988–2002	BAT, MAT, quarantine and movement control
	Brazil	1996	1998–1998	BAT
*Bactrocera dorsalis* Oriental fruit fly *	Rota Island, USA	unknown	1962–1963	MAT (methyl eugenol and naled impregnated cane fibre squares)
	Guam	unknown	1963–1965	SIT
	Mariana Islands, USA	unknown	1964–1964	MAT and SIT
	Kikajima Island, Japan	1929	1968–1978	MAT
	Amami Islands, Japan	1946	1974–1978	MAT
	San Diego, California, USA	1974	1974–1975	MAT, BAT, quarantine and movement control,
	Okinawa Islands, Japan	1919	1977–1985	SIT
	Mauritius	1996	1996–1998	Destruction of infested fruit, MAT, BAT, cover sprays, soil drenching [134]
	Nauru	1992	1998–1999	MAT (fipronil and methyl eugenol)
	Queensland, Australia (Cairns (primary incursion site), Mt Isa, Humpty Do and Darwin)	1995–1997	1995–1999	Quarantine and movement control, MAT (fibreboard blocks with methyl eugenol and malathion), BAT (malathion)
	Torres Strait Islands, Australia	Ongoing seasonal incursions [18]	Multiple programs 1996–ongoing [18]	MAT (caneite blocks laced with malathion), BAT (malathion or spinosad) [18]
	Florida, USA	1964 ongoing (multiple minor incursions) [122]	Multiple programs 1995–2016	MAT (methyl eugenol and naled), soil drench (diazinon and later lambda-cyhalothrin), host removal and destruction, BAT (spinosad), quarantine and movement control [122]
	California, USA (multiple locations in Los Angeles and Sacramento)	2006–2013	Multiple programs 2006–2013	MAT, quarantine, and movement control
	Easter Island	2010	2010–2011	BAT (spinosad), MAT, destruction of infested fruit
	South Africa (multiple locations)	2010	2010–2011	MAT (fibreboard blocks with methyl eugenol and malathion), BAT (GF-120 and LokLure with malathion), orchard sanitation
*Bactrocera tryoni * Queensland fruit fly	Easter Island, Chile	1971	1972	BAT (hydrolysed protein and malathion), MAT (cuelure and malathion impregnated cordelito)
	South Australia, Australia (multiple urban sites–re-occurring minor incursions from NSW)	1987–1994	Multiple programs 1987–1994	BAT (maldison with protein attractant), removal and destruction of fruit, cover spray (fenthion)
	Perth, Western Australia, Australia	1989, 2011	1989–1990, 2011	BAT (spinosad, Naturalure), MAT and SIT
	Cook Islands	2001	2001–2003	Destruction of infested fruit, quarantine and movement control, BAT
	Ti Tree, Northern Territory (re-occurring minor incursions)	2000–2009	Multiple programs 2000–2009	BAT (spinosad and malathion baits), cover sprays, removal and destruction of infested fruits
	South Australia, Australia (multiple urban sites–re-occurring minor incursions from NSW)	2002–2014	Multiple programs 2002–2014	Removal and destruction of fruit, BAT (maldison with protein attractant) and SIT, black plastic on soil under trees to break lifecycle
	Auckland, New Zealand	2015, 2019	2015, 2019	Quarantine and movement control, host collection and disposal, MAT (methyl eugenol and dichlorvos) and BAT (fipronil or spinetoram) [135]
*Ceratitis capitata* Mediterranean fruit fly	New Zealand (multiple sites)	1906, 1907 and 1996	Multiple programs 1906–1907, 1996	Presumed to be fruit destruction and soil drenches of kerosene (early eradications). BAT (malathion), host removal and destruction.
	Bermuda	1907	1907–1963	BAT (malathion and protein attractant), ground application of dieldrin, MAT baits with trimedlure and dichlorvos
	Florida (multiple locations), USA	1929 ongoing (re-occurring minor incursions)	Multiple programs 1930–2011	Ground application of arsenical molasses followed by copper carbonate, host removal and destruction, BAT (malathion and later spinosad), soil drench of dieldrin and later on diazinon and spinosad, quarantine and movement control
	Texas, USA	1966	1966	BAT (malathion and protein attractant)
	South Australia, Australia (multiple locations)	1968 ongoing (re-occurring minor incursions)	Multiple programs 1968–2019	BAT (malathion and later spinosad), SIT, cover spray (fenthion) and ground drench (chlorpyrifos and later lambda-cyhalothrin), quarantine zone, removal and destruction of fruit (treatment with malathion)
	California, USA (multiple locations)	1975 ongoing (re-occurring minor incursions)	Multiple programs 1975–2006	BAT (malathion and later spinosad), SIT, host removal and destruction, quarantine, and movement control
	Alice Springs, Northern Territory, Australia	1976, 1981 & 1986	Multiple programs 1976–1986	BAT (malathion), host removal and destruction, cover sprays of trichlorfon, fenthion (for destroyed fruit) and dimethoate, MAT (trimedlure and dichlorvos)
	Guatemala (multiple locations)	1977	1977–2013	BAT (malathion and later spinosad), SIT
	Chile (multiple locations)	1963 ongoing (re-occurring minor incursions)	Multiple programs 1990–2013	BAT (malathion and spinosad), SIT, host removal and destruction, quarantine and movement control, soil drench
	Tacna, Peru	1957	1990–2007	SIT
	Argentina (multiple locations)	1992 and 1997	Multiple programs 1992–2012	BAT, host removal and destruction, SIT, cover spray, quarantine and movement control
	Belize	1987	1997–2001	BAT (malathion and later spinosad)
	Managua, Nicaragua	1994	2004–2012	BAT, quarantine and host removal
	Dominican Republic	2015	2015–2017	SIT
*Zeugodacus cucurbitae* melon fly	Rota Island, United States	unknown	1962–1963	SIT
	Southwestern Islands, Japan	1972	1972–1993	SIT [136]
	Nauru	1992, 2001	1998–2001	MAT (caneite blocks with methyl eugenol/fipronil) and BAT (fipronil)
	Kern County, California, United States	unknown	2010–2011	MAT

All data are summarised from the Global eradication and response database (GERDA) [133] unless otherwise stated. * Includes data from *Bactrocera dorsalis* synonymy.

## Data Availability

No new data were created or analyzed in this study. Data sharing is not applicable to this article.
